# A Dietary Supplement Supporting the Resolution of Acute Diarrhea in Dogs

**DOI:** 10.3390/vetsci13080837

**Published:** 2026-08-20

**Authors:** Francesca Perondi, Natascia Bruni, Raffaella Adami, Giorgia Meineri, Lucia Zema, Alice Melocchi, Elisa Martello

**Affiliations:** 1 Department of Veterinary Science, University of Pisa, 56122 Pisa, Italy; f.perondi87@gmail.com; 2 Candioli S.r.l., 10092 Turin, Italy; natascia.bruni@candioli.it (N.B.); raffaella.adami@candioli.it (R.A.); 3 Department of Veterinary Sciences, University of Turin, 10095 Turin, Italy; giorgia.meineri@unito.it; 4 PhormulaMI Lab, Department of Pharmaceutical Sciences, University of Milan, 20133 Milan, Italy; lucia.zema@unimi.it (L.Z.); alice.melocchi@unimi.it (A.M.); 5 Centre for Evidence Based Healthcare, School of Medicine, University of Nottingham, Nottingham NG5 1PB, UK

**Keywords:** dog, supplement, diarrhea, probiotics

## Abstract

(1) Background: Acute diarrhea is a frequent clinical manifestation in dogs, often involving dysbiosis and mucosal barrier impairment. This study evaluated the efficacy of Florentero Complex^®^ (Candioli S.r.l.), a multi-targeted dietary supplement combining probiotics, prebiotics, and antioxidant components, in supporting the recovery of dogs with acute diarrhea. (2) Methods: In a randomized, controlled trial, 28 client-owned dogs presenting with acute diarrhea for 3–4 days were assigned to either a Treated Group (TRT: standard commercial diet plus supplement) or a Control Group (CTR: standard commercial diet alone) for 7 days. Daily defecation frequency (nDEF), fecal score (FS), and inflammatory markers (fecal cytology and C-reactive protein) were monitored throughout the study. (3) Results: Both groups showed a reduction in nDEF over time, with no significant difference observed between groups. However, a significant intergroup difference in FS was observed, with the supplemented dogs showing a faster and more pronounced improvement in stool consistency than the CTR. Fecal cytology and serum CRP levels also showed a trend toward reduced inflammation in the TRT group. (4) Conclusions: Florentero Complex^®^ appears to provide nutritional support that improves clinical signs and stool quality beyond standard dietary management alone.

## Simple Summary

Acute diarrhea is one of the most frequent clinical challenges in veterinary practice, often leading to intestinal imbalance and delaying recovery. This study evaluated whether adding the dietary supplement Florentero Complex^®^ (Candioli S.r.l., which contains selected probiotics, a prebiotic and nutraceutical components) to standard dietary management could accelerate recovery. Twenty-eight dogs with acute diarrhea were randomly assigned to two groups: one received a standard commercial diet alone, while the other received the same diet plus the dietary supplement. The findings suggested a significant overall improvement in clinical signs, driven primarily by a faster restoration of fecal consistency in treated patients. Moreover, a positive trend toward reduced markers linked to gut inflammation was observed. In conclusion, the tested dietary supplement provides effective nutritional support that speeds up clinical recovery beyond dietary management alone.

## 1. Introduction

The management of acute diarrhea in dogs represents one of the most frequent clinical challenges in daily veterinary practice. Although many cases are self-limiting, disruption of the intestinal microbiota, known as dysbiosis, together with impairment of the intestinal mucosal barrier can significantly delay recovery and worsen clinical manifestations [1,2,3]. In this situation, the use of complementary feeds formulated to support microbiota stability and to promote the restoration of physiological digestive function has become fundamental in new therapeutic strategies [4].

The use of probiotics in dietary supplements for companion animals has been documented to exert beneficial effects in cases of dysbiosis and intestinal disorders [1,4,5].

The therapeutic rationale for incorporating probiotics lies in their ability to displace pathogens through competitive exclusion, produce antimicrobial metabolites, and modulate the host’s local immune response [6]. For example, *Saccharomyces cerevisiae* var. boulardii (strain DSM 34246) is a probiotic yeast documented for its efficacy as a gut health stabilizer in dogs, demonstrating positive effects on fecal parameters and immune modulation [7,8]. *S. boulardii* acts by suppressing the growth of pathogens such as *Staphylococcus*, *E. coli*, and *Salmonella*, neutralizing bacterial toxins, and enhancing immune defenses by stimulating secretory IgA [9]. At the same time, *Lactobacillus acidophilus* (CECT 4529), a lactic acid-producing bacterium commonly used in both human and veterinary nutrition, acts as an early colonizer that limits the proliferation of harmful microorganisms like *Clostridium perfringens* through competitive exclusion and modulation of microbial metabolism [10,11]. *L. acidophilus* supports digestive efficiency and may contribute to the modulation of intestinal inflammatory responses [12]. Complementing these effects, *Enterococcus faecium* is a naturally occurring gut bacterium that helps maintain the normal intestinal microbiota, protects against harmful microbes and diarrhea, and exerts effects at both mucosal and systemic levels [4]. However, modern nutritional interventions increasingly favor a multi-targeted approach.

The inclusion of prebiotics in supplements for pets, such as fructooligosaccharides (FOS) or mannan-oligosaccharides (MOS), provides a selective substrate that promotes the growth of beneficial indigenous bacteria, such as *Lactobacillus* and *Bifidobacterium* spp., thereby enhancing the efficacy of exogenous probiotic strains [13]. By providing this targeted nourishment, FOS contribute to the stabilization of the intestinal microbial ecosystem and enhance the overall efficacy of the probiotic strains [4].

Furthermore, the incorporation of additional molecules providing antioxidant support, such as those derived from *Malus domestica*, may offer synergistic effects that help address the multifactorial nature of gastrointestinal disease. Apple-derived fractions are known to neutralize reactive oxygen species (ROS), thereby reducing cellular damage associated with gastrointestinal inflammation and facilitating tissue repair. In addition, Malus domestica represents a significant source of polyphenols and functional fibers, which are widely recognized for their antioxidant activity and mucosa-protective properties. The therapeutic potential therefore lies in the synergistic interaction of these components, contributing to the restoration of intestinal homeostasis [14].

Other functional elements may further support intestinal health. Among them, zinc, an essential trace element involved in numerous enzymatic and immune pathways, has been widely recognized for its role in maintaining intestinal barrier integrity. Zinc contributes to epithelial tissue repair, modulation of mucosal immune responses, and the preservation of normal intestinal barrier function [15].

The primary objective of this study is to determine whether the administration of the dietary supplement Florentero Complex^®^ (Candioli S.r.l.), in addition to standard dietary management, results in a faster resolution of diarrhea compared with standard dietary management alone in dogs with acute diarrhea of 3–4 days’ duration. Florentero Complex^®^ (Candioli S.r.l.) is a formulation containing selected probiotics, a prebiotic and nutraceutical components designed to support intestinal health and promote the restoration of normal digestive function in companion animals.

## 2. Materials and Methods

### 2.1. Study Design

This is a randomized, controlled clinical study conducted in two groups involving client-owned dogs presented with acute diarrhea:

- Treated Group (TRT): standard clinical management (e.g., commercial diet) and Florentero Complex^®^ (Candioli S.r.l., Turin, Italy)
- Control Group (CTR): standard clinical management only

Animals were recruited consecutively as they presented with clinical disease and met the inclusion criteria. Animals were randomly assigned to treatment groups (1:1 ratio) upon recruitment using a computer-generated random sequence. Despite random allocation, chance of baseline imbalances between groups can be observed for certain demographic and clinical variables.

Each subject was followed for an observation period of 7 days.

Client-owned dogs provided written informed consent prior to participation. The experimental protocol was approved (Prot.n. 0454094) by the Ethical Committee of the Department of Veterinary Sciences, University of Turin (Italy).

Dogs were included in the study if they met all of the following criteria:

- Dogs of any breed and sex, aged >12 months
- Acute diarrhea with onset within the previous 3–4 days [16]
- Up to approximately 6 diarrheic episodes/day during the 24 h prior to enrollment
- General condition: good to moderately compromised, without signs of severe dehydration or shock
- No clinical suspicion of surgical abdominal disease (e.g., obstruction, intussusception)
- Owner consent and willingness to follow the study protocol and to complete a daily diary
- Dogs were excluded from the study if they presented with: diarrhea lasting >14 days (chronic diarrhea), evidence of severe systemic disease (e.g., advanced renal or hepatic failure, neoplasia, infection), parasitosis, treatment with other probiotics or systemic antibiotics within the 7 days prior to enrollment, pregnancy or lactation.

### 2.2. Diet and Supplementation

All enrolled dogs received standard management, consisting of a commercial diet (Monge Natural Superpremium Adult Chicken). The use of other probiotics or specific antidiarrheal medications was not allowed, unless clinically required, in which case the subject was withdrawn from the study protocol. Dogs assigned to Group TRT received, in addition to standard management, the supplement administered at the manufacturer’s recommended dose (in tablets), at a dose of 1 tablet (1.2 g) for every 10 kg body weight (BW), once a day for a treatment duration. Dogs assigned to Group CTR received the standard diet, without administration of the supplement or any other probiotic products.

Although the tested product was supplied by the manufacturer, potential bias was minimized through random allocation of animals to treatment groups, independent clinical assessments by a veterinarian unaffiliated with the manufacturer, independent laboratory analyses and data analysis, all of which were conducted according to the pre-specified study protocol.

### 2.3. Data Collection

At Day 0 (T0, enrollment), signalment data for each dog, including breed, sex, age, BW and body condition score (BCS), were recorded, and a complete medical history was collected, covering the onset of diarrhea, diet, and any previous treatments. A full general clinical examination was performed by the same veterinarian. Diarrhea was evaluated by recording the number of episodes in the previous 24 h (nDEF) and assessing fecal consistency using a numerical scoring scale (fecal score, FS: 1–7, Nestlè Purina, PetCare, St. Louis, MO, USA) [17]. Hematochezia (EMAT) and flatulence/meteorism (FLATMET) were recorded as present or absent.

In the laboratory, blood and fresh fecal samples were collected and tested, respectively, for serum C-reactive protein (CRP) and fecal cytology (assessing mono-/pleomorphism, neutrophil presence, and bacterial phagocytosis) by the same operator. Serum CRP determination was performed using an IDEXX Catalyst Dx automated biochemistry analyzer (IDEXX Laboratories, Inc., Westbrook, ME, USA), employing immunoturbidimetric/enzymatic methodology according to the applicable analytical panel.

At enrollment, owners were provided with a daily clinical diary sheet and instructed on its completion. The completed diary was collected and included in the clinical file of each subject. Owners were asked to record the nDEF, FS, EMAT, FLATMET, presence of mucus, vomiting, appetite, and any adverse event. The owners were trained by the veterinarian at T0 to be able to record data appropriately in the diary. These parameters were used for the overall clinical assessment. According to the study protocol, symptom resolution was defined as achievement of FS = 3 and fewer than 2 defecations per day.

Follow-up assessments were performed at Day 2 (T1) and Day 4 (T2), either during a clinical visit or via telephone contact, with an additional visit if required. During these evaluations, the veterinarian reviewed the owner’s diary, assessed the number of nDEF, FS, EMAT, and FLATMET as recorded by the owner, and noted an overall clinical judgment of improvement (improved, unchanged, or worsened). The overall clinical judgment refers to the integrated evaluation of these clinical parameters over time.

At Day 7 (T3), a final clinical evaluation was conducted by the same veterinarian assessing the same clinical and laboratory parameters as at T0.

### 2.4. Statistical Analysis

The statistical analysis was performed using R software (version 4.3.2; R Foundation for Statistical Computing, Vienna, Austria), and the statistical approach was selected according to the characteristics of each variable analyzed.

For the nDEF, a generalized linear mixed model (GLMM) for repeated measures was applied. For the ordinal FS (1–7 scale), a cumulative link mixed model was applied. For longitudinal binary clinical endpoints (hematochezia, EMAT; flatulence/meteorism, FLATMET), initial standard logistic mixed models failed to converge due to complete data separation. To overcome separation bias and ensure stable model estimation, Firth’s penalized likelihood logistic regression was applied. All mixed models included subject as a random effect and treatment group, time, and treatment × time interaction as fixed effects, in order to account for within-subject correlation and inter-individual variability [18,19]. The model was defined as follows:

y = μ + S_i_ + G_j_ + T_k_ + GT_jk_ + e_ijkn_

- y—dependent variable (the observed outcome)
- μ—overall mean of the dependent variable
- S_i_—random effect of the *i*-th subject (captures individual variability)
- G_j_—fixed effect of the *j*-th treatment (differences due to treatment groups)
- T_k_—fixed effect of the *k*-th time point (differences due to time)
- GT_jk_—fixed effect of the interaction between treatment *j* and time *k* (how treatment effects change over time)
- e_ijkn_—residual error for the *n*-th observation of subject *i*, treatment *j*, at time *k* (unexplained variation)

Fecal cytology (mono-/pleomorphism, neutrophil presence, and bacterial phagocytosis) was analyzed between groups using Fisher’s exact test [20].

For CRP, the Mann–Whitney U test was applied because data were not normally distributed [21].

Overall time to clinical resolution (number of days required to achieve complete symptom resolution, defined as FS = 3 and nDEF < 2) was compared between groups (CTR vs. TRT) using the non-parametric Mann–Whitney U test [21], as data were discrete and non-normally distributed. Data for time to resolution are expressed as median [interquartile range, IQR (Q1–Q3)].

Treatment effect sizes with 95% Confidence Intervals [95% CI] were estimated using post-hoc pairwise contrasts: Rate Ratios (RR) for count data, Odds Ratios (OR) for ordinal/binary GLMMs and Fisher’s tests, Risk Differences (RD, %) for bacterial phagocytosis, and Hodges–Lehmann median differences for CRP. A *p*-value < 0.05 was considered statistically significant.

## 3. Results

A total of 28 dogs presenting with diarrhea for the past 3–4 days were enrolled and divided into two groups: the control group (CTR) and the treatment group (TRT), which received Florentero Complex^®^ (Table 1) in addition to standard management.

Signalment data for the two groups is summarized in Table 2. All enrolled dogs were mixed-breed animals, and no breed-specific stratification was applied in the study design or statistical analysis. None of the dogs had mucus in the feces or vomiting, and all maintained their appetite. No adverse events were recorded.

Analysis reported in Table 3 revealed that, for nDEF, a main effect of time (T: *p* = 0.0224), indicating a reduction in nDEF over the study period in both groups. However, no significant differences were detected between groups (G: *p* = 0.1500; G × T interaction: *p* = 0.8728), and the estimated Rate Ratios at all-time points spanned 1 (Table 3), indicating that defecation frequency did not differ significantly between CTR and TRT at any time point.

However, for FS, the model demonstrated a significant main effect of time (*p* < 0.0001) and a trend for the group × time interaction (*p* = 0.0780). Post-hoc pairwise contrasts revealed a statistically significant difference between groups at T3 (*p* < 0.05; OR = 6.78 [1.38–33.30]), with the TRT group exhibiting a significantly greater improvement in stool consistency compared with the CTR group at T3 (Table 3).

Moreover, a significant main effect of time was observed for EMAT. In particular, it resolved completely by T2 and remained absent at T3, whereas isolated cases persisted in the control group. However, no significant main effect of group or group × time interaction was observed, indicating no statistically significant difference in EMAT resolution between CTR and TRT.

For FLATMET, penalized logistic regression revealed a highly significant overall time effect reflecting a marked decrease in symptoms over the study period in both groups. No significant group effect or group × time interaction was observed, indicating that FLATMET resolved at a similar rate in both control and supplemented dogs (Table 3).

No other parameters described in Table 3 showed statistically significant differences.

Regarding fecal cytology, a reduction in inflammatory markers was observed in the TRT group between T0 and T3. At baseline, 8 subjects showed neutrophil presence, and 7 exhibited bacterial phagocytosis; by T3, these cases decreased to 2 and 0, respectively (Table 3). In contrast, the CTR group maintained a higher number of subjects with neutrophils at the final time point (Table 3).

All the enrolled dogs achieved full clinical resolution (FS = 3 and fewer than 2 defecations per day) during the study period. The overall time required to achieve complete resolution did not differ significantly between groups (*p* = 0.3771). Median time to resolution was 2.5 days [IQR: 1.0–4.0 days] in the CTR group and 2.0 days [IQR: 1.0–3.0 days] in the TRT group.

## 4. Discussion

Acute gastrointestinal disease in dogs, including acute diarrhea, is one of the most common clinical presentations in small animal veterinary practice. Although many cases resolve quickly and with no consequences, disturbances of intestinal health together with impairment of the intestinal mucosal barrier can contribute to delayed recovery and increased severity of clinical signs [1,3,16]. Similar pathophysiological mechanisms may also be involved in chronic enteropathies, in which periods of clinical stability can be interrupted by episodes of acute worsening or recurrent diarrhea, further supporting the importance of strategies aimed at restoring gastrointestinal homeostasis [22,23,24].

For this reason, nutritional strategies aimed at restoring gastrointestinal homeostasis may be valuable not only during acute diarrheic episodes, but more broadly in dogs presenting with diarrhea of different origin or duration. In this context, Florentero Complex^®^, through the combination of probiotics, prebiotics, antioxidant compounds, and supportive nutrients, has been developed to support intestinal health, mucosal integrity, and physiological digestive function in dogs with diarrhea, irrespective of whether the condition is acute, recurrent, or associated with chronic gastrointestinal imbalance [2,4,5].

The results of this clinical study in dogs with acute diarrhea demonstrated that the tested supplementation led to a significant overall improvement in clinical recovery, driven primarily by a faster restoration of fecal consistency. Interestingly, at the final time point, FS in the TRT group did not exceed the target score of 3, even when considering variability, whereas some dogs in the CTR group remained close to a score of 4. By T3, acute diarrheic episodes had improved in both groups, consistent with the self-limiting nature of acute, non-severe diarrhea; however, the significantly lower FS in the TRT group at T3 indicates a sustained benefit and a more complete normalization of stool consistency by the end of the study period. Supplemented dogs showed a slight descriptive trend toward earlier resolution of nDEF, although no statistically significant between-group difference was observed. This may suggest a potential beneficial effect of supplementation that warrants further investigation in larger studies. These findings are consistent with the proposed rationale for the use of complementary feeds aimed at supporting intestinal stability and promoting the restoration of normal digestive function. It is noteworthy that clinical improvement was also observed in the control group, albeit over a longer time course compared with treated animals. This clinical improvement is likely attributable to the administration of a controlled commercial diet, with strict avoidance of snacks or any additional food outside of meals provided by the owner. Such dietary consistency helps reduce gastrointestinal variability and is a key factor in resolving acute diarrhea. However, the faster resolution of clinical signs in the TRT group suggests that nutraceutical supplementation exerts an additional synergistic effect, accelerating recovery beyond dietary management alone. The enhanced clinical response observed in the TRT group may be explained by a synergistic interaction among its components, particularly the combination of probiotics and prebiotics. The inclusion of *S. cerevisiae* var. boulardii (DSM 34246) and *L. acidophilus* (CECT 4529) may help counteract intestinal dysbiosis through competitive exclusion of pathogenic microorganisms and modulation of the local immune response [7,8,9]. The activity of these probiotic strains may have been further supported by prebiotic substrates such as fructo-oligosaccharides (FOS), which selectively promote the growth of beneficial commensal bacteria [4,13]. As previously reported in the literature [11], supplementation with targeted probiotic strains such as *L. acidophilus* D2/CSL, when integrated into a multifactorial nutritional strategy, provides measurable benefits in canine health and nutritional status. These findings further support the concept that the combined use of probiotics, prebiotics, and supportive nutraceuticals may be more effective in restoring normal gastrointestinal function than dietary management alone. A critical aspect of the recovery process involves addressing the gastrointestinal (GI) tract as a major site for the generation of pro-oxidants. In GI diseases, oxidative stress arises from an imbalance between microbial-elicited pro-oxidants and antioxidant defenses. Consequently, attention has increasingly focused on the role of antioxidant supplementation during acute diarrheic episodes [16]. In this trial, the integration of the Vexantra^®^ complex, derived from *Malus domestica* (apple) together with zinc glycinate chelate (with zinc not administered in elemental form), may have contributed to supporting intestinal mucosal physiology in the treated dogs. However, the observed clinical effects cannot be specifically attributed to these individual components, as they may represent one of several complementary nutritional mechanisms contributing to the overall effects of the formulation. Adequate zinc availability contributes to epithelial tissue repair, modulation of mucosal immune responses, and preservation of normal intestinal barrier function [15,25].

Dietary active compounds such as polyphenols exert a protective role against GI diseases by modulating inflammation-related cellular events and the composition of microbiota populations [14,16]. *Malus domestica* fractions may neutralize reactive oxygen species (ROS), thereby reducing cellular damage and supporting tissue repair [14].

Acute mucosal erosion can result in the presence of blood in feces during the initial phases of diarrhea. In the present study, supplementation with the tested product was accompanied by a rapid and complete resolution of hematochezia as early as T2. EMAT and FLATMET, considered secondary clinical outcomes, showed significant progressive resolution over time across the entire study cohort. However, no statistically significant differences between the CTR and TRT groups were observed at any time point for either parameter. Nevertheless, the rapid resolution of hematochezia observed in the supplemented group represents a favorable clinical trend, although it cannot be conclusively attributed to the treatment. Overall, these findings indicate that both clinical manifestations improved rapidly as the acute enteric condition resolved.

Although fecal cytology did not reach statistical significance (Table 3), the descriptive data and estimated effect sizes suggested a progressive improvement in the TRT group compared with the CTR group. The complete resolution of bacterial phagocytosis, together with the numerical decrease in the proportion of subjects exhibiting mucosal neutrophil infiltration and pleomorphism in the TRT group by T3, provides supportive evidence of a favorable anti-inflammatory trend at the intestinal mucosal level. Although these changes cannot be conclusively attributed to the supplementation, they may indicate a potential beneficial effect of the nutraceutical intervention and suggest that it could contribute to the restoration of mucosal homeostasis.

These observations are supported by one study using fecal cytology as a useful diagnostic component for evaluating gastrointestinal inflammation in dogs. While neutrophils are typically absent or found in very low numbers in clinically healthy subjects, their presence in fecal smears is considered an indicator of mucosal involvement [26]. Therefore, the numerical reduction in neutrophil counts and the resolution of bacterial phagocytosis observed in the TRT group may provide supportive evidence of a decrease in the inflammatory state. A similar trend was observed for CRP levels, with the TRT group showing a tendency to decrease in mean values (Table 3). This clinical trend could be attributed to a reduction in systemic inflammation following the stabilization of the intestinal environment. As demonstrated by Jergens et al. (2003) [27], serum CRP concentrations significantly correlate with disease activity in dogs with inflammatory bowel disease (IBD) and serve as a reliable laboratory marker to evaluate the effect of a therapy. Our results, while not statistically significant due to the small sample, appear to be consistent with results reported in Jergens et al. (2003) [27], where a reduction in CRP levels is often associated with clinical improvement following medical intervention.

We acknowledge that the absence of blinding and a placebo control may have introduced bias, particularly for subjective outcomes such as FS, although assessments were performed according to predefined criteria to promote consistency. While the primary clinical endpoints improved, secondary measures, including serum CRP, fecal cytology, EMAT, and FLATMET, did not reach formal statistical significance between groups. This may partly reflect the substantial inter-individual physiological variability inherent to these secondary markers, together with the rapid overall clinical recovery observed in both cohorts, which may have limited the ability to detect between-group differences. Nevertheless, the descriptive data showed favorable and consistent trends in several parameters in the supplemented dogs, including indicators of inflammation and mucosal recovery. Although these observations cannot be considered evidence of a statistically confirmed treatment effect, they may indicate a potential beneficial biological influence of supplementation. Additional limitations include the reliance on owner-reported outcomes and the absence of an etiological investigation to determine the underlying cause of diarrhea. These factors should be considered when interpreting the findings and highlight the need for larger, blinded, placebo-controlled studies to further evaluate the potential benefits of the supplementation.

## 5. Conclusions

Taken together, the findings of the present study indicate that Florentero Complex^®^ (Candioli S.r.l.) may represent an effective nutritional support for dogs with diarrhea, contributing to a significant improvement in stool consistency and resolution of clinical signs compared with dietary management alone. Although the clinical trial specifically evaluated dogs with acute diarrhea, the multimodal characteristics of the formulation suggest potential usefulness in a broader range of diarrheic conditions in which intestinal dysbiosis, impaired mucosal barrier function, or local inflammatory imbalance may be involved.

Through the combined action of targeted probiotics, prebiotics, antioxidant compounds, and supportive nutrients, Florentero Complex^®^ may contribute to the restoration of intestinal health and the maintenance of mucosal integrity. The favorable trends observed in several secondary clinical and biological parameters, although not statistically significant between groups, further support the potential complementary role of the formulation in intestinal recovery. Therefore, future studies evaluating the same product in larger, controlled populations may help clarify its potential benefits in recurrent or chronic gastrointestinal disturbances, during periods of gastrointestinal stress, and during or following antibiotic treatments, when disruption of the intestinal ecosystem is commonly observed.

## Figures and Tables

**Table 1:** Percentage (%) and ingredient composition of Florentero^®^ Complex (Candioli S.r.l.).

Florentero^®^ Complex (Candioli S.r.l.)	
Ingredients	%
Probiotics: Saccharomyces cerevisiae DSM 34246 (Boulardii, 1.35 × 10^13^ CFU/kg), Lactobacillus acidophilus D2/CSL (1.6 × 10^11^ CFU/kg), Enterococcus faecium DSM10663/NCIMB 10415 (9.8 × 10^11^ CFU/kg)	8.10
Palatability enhancer	2.00
Prebiotics and Yeast Derivatives: Fructooligosaccharides (FOS), Mannanoligosaccharides (MOS), Yeast Products (Epicorpets)	49.93
BacNutra (bioactive peptide blend)	2.00
Vexantra^®^ (plant-derived nanovesicles associated with zinc glycinate chelate; 4736.88 mg/kg zinc glycinate chelate, corresponding to 72.47 mg/kg in the final product)	1.53
Vitamins: B1, B2, B6, B12, E (4958.65 mg/kg)	0.51
Magnesium Sulfate Heptahydrate, Sodium Chloride, Potassium Sulfate	0.61
Excipients and Tableting Aids: Magnesium Stearate, Compritol E, Colloidal Silica, Microcrystalline Cellulose, Prosolv SMCC 90, Dicalcium Phosphate Dihydrate, Corn Starch	35.22
Emulsifiers: Soy Lecithin	0.10
Total	100.00

**Table 2:** Signalment data of the enrolled dogs. Data are expressed as number of subjects for sex and as mean ± standard deviation (SD) for age, weight, body condition score (BCS), and duration of diarrhea.

	CTR (*n* = 14)	TRT (*n* = 14)
Sex (M, F)	10 M, 4 F	7 M, 7 F
Age (years)	5.4 ± 4.1	3.8 ± 3.4
Weight (kg)	17.5 ± 17.8	15.3 ± 8.8
BCS (1–9)	5.2 ± 1.5	4.8 ± 1.2
Duration of diarrhea (days)	3.8 ± 0.9	3.9 ± 1.2

**Table 3:** Primary clinical outcomes, laboratory markers, and estimated treatment effects across study timepoints in Control (CTR, *n* = 14) and Treated (TRT, *n* = 14) groups. Data are presented as mean ± standard deviation for daily defecation frequency (nDEF); median [interquartile range, IQR (Q1–Q3)] for fecal score (FS) and C-reactive protein (CRP); count (percentage, %) of positive subjects for hematochezia (EMAT), flatulence/meteorism (FLATMET), cytology (mono-/pleomorphism, neutrophil presence, and bacterial phagocytosis).

Parameter	Group	T0	T1	T2	T3	*p*-Value	Effect Size [95% CI]
nDEF	CTR	5.1 ± 2.0	3.7 ± 1.0	2.8 ± 1.0 *	2.5 ± 0.8 *	G: *p* = 0.1500	T0: 1.29 [0.91–1.81] ^1^
	TRT	4.0 ± 1.3	2.9 ± 1.3	2.6 ± 1.3	2.3 ± 1.0 *	T: *p* = 0.0224	T1: 1.30 [0.86–1.96] ^1^
						G × T: *p* = 0.8728	T2: 1.08 [0.69–1.69] ^1^
							T3: 1.09 [0.67–1.78] ^1^
FS	CTR	4 [3.25–5]	4 [2.25–4]	3 [3–3.75]	4 [4–4] ^a^	G: *p* = 0.5017	T0: 0.55 [0.10–3.10] ^2^
	TRT	4.5 [4–5]	4 [2–4]	2 [2–3]	3 [2.25–3] ^b^	T: *p* < 0.0001	T1: 1.45 [0.28–7.58] ^2^
						G × T: *p* = 0.0780	T2: 4.44 [0.90–21.94] ^2^
							T3: 6.78 [1.38–33.30] ^2^
EMAT	CTR	7 (50%)	3 (21.4%)	1 (7.1%)	1 (7.1%)	G: *p* = 0.7143	T0: 0.76 [0.02–3.23] ^2^
	TRT	6 (42.9%)	1 (7.1%)	0 (0.0%)	0 (0.0%)	T: *p* = 0.0135	T1: 0.48 [0.031–5.52] ^2^
						G × T: *p* = 0.6079	T2: 0.41 [0.002–11.39] ^2^
							T3: 0.41 [0.002–11.39] ^2^
FLATMET	CTR	14 (100.0%)	10 (71.4%)	3 (21.4%)	2 (14.3%)	G: *p* = 0.2054	T0: not estimable ^2^
	TRT	12 (85.7%)	9 (64.3%)	2 (14.3%)	1 (7.1%)	T: *p* < 0.0001	T1: 4.29 [0.20–713.29] ^2^
						G × T: *p* = 0.5309	T2: 3.81 [0.15–672.04] ^2^
							T3: 3.22 [0.09–618.54] ^2^
Mono-/pleomorphism	CTR	1 (7.1%)	-	-	1 (7.1%)	T0: *p* = 0.9990	T0: 2.17 [0.17–50.8] ^2^
	TRT	2 (14.3%)	-	-	0 (0.0%)	T3: *p* = 0.9990	T3: 0.00 [0.00–37.9] ^2^
Neutrophil presence	CTR	6 (42.9%)	-	-	4 (28.6%)	T0: *p* = 0.704	T0: 1.78 [0.39–8.16] ^2^
	TRT	8 (57.1%)	-	-	2 (14.3%)	T3: *p* = 0.6483	T3: 0.42 [0.06–2.91] ^2^
Bacterial phagocytosis	CTR	3 (21.4%)	-	-	0 (0.0%)	T0: *p* = 0.245	T0: 28.6% [−6.5–63.7] ^3^
	TRT	7 (50.0%)	-	-	0 (0.0%)	T3: *p* = 1.0	T3: 0.0% [−12.5–12.5] ^3^
CRP (mg/L)	CTR	6.4 [4.0–10.8]	-	-	5.2 [3.5–8.4]	T0: *p* = 0.897	T0: 0.60 [−7.10–8.40] ^4^
	TRT	6.5 [3.9–21.7]	-	-	3.6 [1.9–5.2]	T3: *p* = 0.1677	T3: −1.90 [−5.80–2.10] ^4^

^1^ Rate Ratio (RR); ^2^ Odds Ratio (OR); ^3^ Risk Difference (RD, %); ^4^ Hodges–Lehmann median difference (mg/dL). * *p* < 0.05 within the same group compared to T0. ^a,b^ between groups at the same time point (*p* < 0.05). Abbreviations: nDEF, daily defecation frequency; FS, fecal score; EMAT, presence of hematochezia; FLATMET, presence of flatulence/meteorism; CRP, C-reactive protein; CTR, control group; TRT, treated group; CI, confidence interval; G, group effect; T, time effect; G × T, group × time interaction.

## Data Availability

The original contributions presented in this study are included in the article. Further inquiries can be directed to the corresponding author.

## Abbreviations

The following abbreviations are used in this manuscript:

FOS	Fructooligosaccharides
MOS	Mannan-oligosaccharides
IBD	Inflammatory bowel disease
ROS	Reactive oxygen species
nDEF	Number of defecations
EMAT	Hematochezia
FLATMET	Flatulence/meteorism
FS	Fecal score
CRP	C-reactive protein
GI	Gastrointestinal
TRT	Treated
CTR	Control
